# Reducing Postoperative Needling in XEN63^®^ Surgery with Adjunctive Cross-Linked Hyaluronic Acid Implant (Healaflow^®^): Early Evidence from Real-World Practice

**DOI:** 10.3390/jcm14165848

**Published:** 2025-08-19

**Authors:** Pier Luigi Guerin, Gabriella Cirigliano, Gian Marco Guerin, Daniele Tognetto

**Affiliations:** Eye Clinic, Department of Medical, Surgical Sciences and Health, University of Trieste, 34129 Trieste, Italy; gabriellacirigliano16@gmail.com (G.C.);

**Keywords:** glaucoma surgery, XEN63, Healaflow, fibrosis, needling

## Abstract

**Background:** Open-angle glaucoma (OAG) is a leading cause of irreversible blindness. While trabeculectomy remains the surgical gold standard, bleb-forming minimally invasive procedures such as the XEN63 gel stent offer a safer alternative. However, early postoperative management remains critical, as needling is frequently required to preserve bleb function. Healaflow^®^ (HF), a cross-linked hyaluronic acid gel, has been proposed as an adjunct in glaucoma surgery to maintain the subconjunctival space and modulate fibrosis. This study aimed to evaluate the outcomes of XEN63 implantation with or without HF in terms of IOP reduction, glaucoma medication use, surgical success, and postoperative intervention rates. **Methods:** This retrospective, comparative study included 20 pseudophakic eyes with medically uncontrolled OAG undergoing XEN63 implantation with mitomycin-C (MMC), either with (n = 10) or without (n = 10) adjunctive HF. Follow-up included IOP measurements, medication use, and the need for postoperative procedures up to 3 months. **Results:** At 3 months, both groups showed significant IOP reduction from baseline (−31.8% in XENhf vs. −38.8% in XENa, *p* > 0.05) with minimal medication use. Complete success was achieved in 90% of XENhf eyes and 80% of XENa eyes. Although the proportion of eyes requiring needling did not differ significantly, the total number of procedures was lower in the HF group (2 vs. 8; *p* = 0.004). **Conclusions:** Adjunctive HF use in XEN63 surgery may reduce the number of postoperative procedures while maintaining equivalent efficacy and safety, potentially easing the early management burden for both patients and clinicians.

## 1. Introduction

Open-angle glaucoma (OAG) is a leading cause of irreversible blindness, and lowering intraocular pressure (IOP) is the only proven strategy to slow its progression [1]. Trabeculectomy with adjunctive antifibrotic agents like mitomycin-C (MMC) remains the gold-standard glaucoma surgery for achieving a low target IOP [2]. Minimally invasive bleb-forming glaucoma surgeries (MIBSs) have been developed as safer alternatives to traditional filtering surgery, and the XEN63 gel stent (AbbVie, Dublin, Ireland) (XEN63) is one of the widely adopted devices designed to create a subconjunctival bleb through an ab interno approach [3]. However, like in other filtering surgeries, XEN63 blebs are prone to scarring and failure if the subconjunctival fibrosis is not controlled. In real-world series, the need for postoperative needling or revision of XEN63 blebs to restore filtration is commonly reported [3].

To improve bleb outcomes, various wound modulation strategies have been explored in glaucoma surgery beyond MMC. A newer approach is the use of a cross-linked hyaluronic acid gel implant (Healaflow^®^, Aptissen SA, Geneva, Switzerland) as a space-maintaining, antifibrotic adjunct to glaucoma surgery. Healaflow (HF) is a long-lasting biocompatible, viscoelastic gel of cross-linked sodium hyaluronate that can be injected subconjuctivally around the filtration site and has already been used in other glaucoma surgery already [4,5]. Over a period of up to six months, it acts as a physical spacer to maintain an aqueous outflow pathway and a diffuse bleb, while its biochemical properties modulate wound healing. Experimental and clinical studies have shown that hyaluronic acid (HA) can inhibit pro-fibrotic cytokines and inflammation, reduce oxidative stress, and slow fibroblast proliferation and angiogenesis in the bleb, thereby delaying or preventing scar formation. Moreover, thanks to its structural properties, HF promotes a controlled and uniform outflow of aqueous humor, reducing postoperative complications associated with hypotony [5,6].

To date, evidence on using HF with XEN63 implants is very limited. A recent case report described a XEN45 implantation combined with HF in a refractory glaucoma patient, noting that the cross-linked HA helped maintain a diffuse bleb and stable, low IOP without complications [7]. However, no studies have examined adjunctive HF with the newer XEN63 stent. To the authors’ knowledge, our study is the first to compare XEN63 gel stent implantation versus without HF in OAG. We hypothesized that the addition of the cross-linked HA gel would mitigate postoperative fibrosis, leading to more stable, functioning blebs, fewer postoperative interventions such as bleb needling or revision, and improved surgical success.

## 2. Materials and Methods

This study was designed as a retrospective, comparative analysis evaluating the outcomes of XEN63 gel stent implantation with or without the use of HF in patients with OAG. Clinical data of patients who had undergone uneventful XEN63 implantation for medically uncontrolled OAG between April and December 2024 were collected. Eyes with a history of previous ocular surgery (except for uncomplicated cataract extraction) or with secondary glaucoma (other than pseudoexfoliative glaucoma) were excluded. A total of twenty pseudophakic eyes from twenty patients were included and divided into two groups based on the use of adjuvant material: XEN63 alone (XENa, n = 10) and XEN63 combined with HF (XENhf, n = 10). The local ethics committee approved this study, and the tenets of the Declaration of Helsinki were followed throughout. Informed written consent was obtained from each patient. During the preparation of this manuscript, the authors used ChatGPT based on the GTP-4o architecture, 2025 version (OpenAI, San Francisco, CA, USA) to support the revision of English language and enhance the fluency of the text. The authors have reviewed and edited the output and take full responsibility for the content of this publication.

### 2.1. Data Acquisition

Data were collected retrospectively by reviewing the medical records of consecutive patients who underwent XEN63 implantation during the study period. Information regarding preoperative characteristics, intraoperative details, and postoperative outcomes was extracted from electronics charts. Exclusion criteria were a follow-up shorter than 3 months, incomplete clinical data, or any intraoperative complication during surgery. All patients underwent complete ophthalmological examination within one week before the surgery and were followed with examinations at 1 day, 1 week, 1 month, and 3 months, with additional visits and procedures as needed. The following preoperative baseline data were collected: age, gender, best-corrected visual acuity (BCVA), glaucoma type, intraocular pressure (IOP), and the number of prescribed antiglaucoma medications (including oral acetazolamide). At each visit, IOP, measured by Goldmann applanation tonometry; BCVA; and the number of glaucoma medications were recorded. The filtering bleb was evaluated by slit-lamp biomicroscopy, as were any other upcoming complications.

### 2.2. Surgical Technique

All included eyes had undergone XEN63 ab interno implantation by two experienced surgeons (DT, GC) using a standardized, uneventful technique. The choice between topical and peribulbar anesthesia was made according to the surgeon’s preference. At the beginning of each procedure, the superonasal conjunctiva was gently dissected by injecting 0.1 mL of bupivacaine/adrenaline solution. The XEN63 was then implanted via an ab interno approach, and the mobility of the device beneath the conjunctiva was verified by performing sideways movements. In all cases, a 0.1 mL injection of MMC (0.2 mg/mL) was administered into the subconjunctival space posterior to the XEN63. At the end of surgery, eyes in the XENhf group received an injection of cross-linked sodium hyaluronate gel. Using a 27-gauge needle, approximately 0.1 mL was injected uniformly between the Tenon’s capsule and the conjunctiva around and posterior to the XEN implant’s external end, creating a space between the implant and the surrounding conjunctiva, in order to prevent direct contact between the two structures and to maintain the patency of the filtration area. Gentle massage of the conjunctiva with a blunt spatula was then performed to promote uniform spread of the HF and to minimize the risk of localized accumulation. The control group underwent the same XEN63 procedure and MMC application but without any HF injection.

Postoperative management was identical in both groups, including topical antibiotics for 1 week and slowly tapering anti-inflammatory steroid drops over the following months. Needling procedures were performed by the same surgeon (GC) in a class A minor surgery room for eyes presenting with a flattened bleb and an IOP > 18 mmHg measured on two consecutive control visits. In the XENhf group, since the bleb remained consistently well-elevated due to the presence of the hyaluronic acid-based gel, the indication for needling was based solely on IOP elevation. As our clinical practice, needling is considered the first-line treatment in these cases and could be repeated after 1 week if the initial procedure was insufficient. No 5-fluorouracil (5-FU) injections were given postoperatively unless part of a needling procedure. A completely non-draining bleb, repeated unsuccessful needlings, or poorly visible XEN63 implant are considered indications for open bleb revision. If this approach failed or the patient refused to undergo these procedures, topical hypotensive medication was reintroduced.

### 2.3. Study Objectives

The outcomes evaluated in this study included IOP reduction, change in the number of glaucoma medications, the overall surgical success rate, the total number of needling procedures performed within 3 months, and the proportion of eyes undergoing at least one needling. Complete surgical success was defined as achieving a target IOP (≤18 mmHg and ≥20% IOP reduction) without any glaucoma medications, as measured at the final 3-month follow-up visit. Qualified success allowed the use of topical medications to reach the target IOP. Safety outcomes and postoperative complications were also analyzed and compared between the two groups.

### 2.4. Statistical Analysis

Statistical Package for the Social Sciences version 25.0 for Macintosh (SPSS Inc., Chicago, IL, USA; IBM) was used for descriptive and statistical analysis. The normality of data distribution was assessed using the Shapiro–Wilk test. Normally distributed continuous variables were expressed as the mean ± standard deviation (SD), while categorical variables were presented as absolute frequencies and percentages. Intra-group changes in IOP from baseline to follow-up were assessed using paired *t*-tests. Between-group comparisons of continuous variables (IOP, number of medications, BCVA) were performed using either independent *t*-tests or the Mann–Whitney U test, depending on the data distribution. Categorical variables such as the success rate or complication rate were analyzed using Fisher’s exact test. To assess the needling burden, both the proportion of patients undergoing at least one needling procedure and the total number of needling procedures performed were evaluated. The former was compared using Fisher’s exact test, while the latter was analyzed using a two-proportions z-test. A two-sided *p* value < 0.05 was considered statistically significant.

## 3. Results

A total of 20 eyes from 20 patients with open-angle glaucoma were included in the study, with 10 eyes in the XENhf group and 10 eyes in the XENa group. Baseline demographic and ocular characteristics were comparable between the two groups (Table 1).

The mean preoperative IOP was 21.9 ± 2.9 mmHg in the XENhf group and 22.7 ± 3.2 mmHg in the XENa group (*p* = 0.56). Patients in the XENhf group received an average of 3.2 ± 0.9 medications preoperatively, compared to 3.1 ± 0.8 in the XENa group (*p* = 0.81). The distribution of glaucoma types did not differ significantly between groups, with primary open-angle glaucoma being the most common diagnosis, and all patients were pseudophakic.

In the postoperative results (Table 2), both groups achieved significant IOP reductions following XEN63 implantation.

At 3 months postoperatively, the mean IOP in the XENhf group was 14.8 ± 2.9 mmHg, corresponding to a 31.8% reduction, with medication use reduced to 0.1 ± 0.3. In the XENa group, the mean IOP was 13.6 ± 2.1 mmHg, reflecting a 38.8% reduction from baseline, and the average number of medications decreased to 0.2 ± 0.4. The final corrected distance visual acuity (CDVA) was 0.13 ± 0.12 in the XENa group and 0.14 ± 0.11 in the XENhf group. No statistically significant differences were observed between the two groups in any of these 3-month outcomes.

The success rate was similar between the two groups at the final 3-month follow-up visit. By definition, qualified success was achieved in 100% of eyes in both groups, while complete success was achieved in 90% (9/10) of XENhf eyes and 80% (8/10) of XENa. No eye required additional glaucoma surgery within this 3-month follow-up period.

Flattened blebs and raised IOP developed in four control eyes (40%) within 3 months and in two eyes (20%) in the HF group (*p* = 0.63); these cases were managed by bleb needling, while one eye in the XENa group required open bleb revision. Although the percentage of eyes requiring bleb needling or revision did not differ significantly between the groups, the total number of procedures was higher in the XENa group (8 vs. 2). The Mann–Whitney U test comparing the number of procedures between the two groups did not reach statistical significance (*p* = 0.243) while a two-proportions z-test revealed a significant difference in total procedure frequency (*p* = 0.004), indicating a substantially higher postoperative intervention burden in the XENa group. This reflects the fact that patients in the XENa group often required multiple procedures to restore bleb function, whereas in the XENhf group, a single intervention was generally sufficient to achieve sustained IOP control.

**Figure 1:**
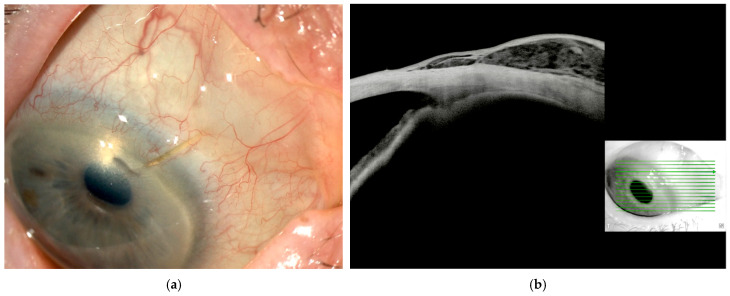
Anterior segment photo (**a**) and anterior segment-optical coherence tomography (AS-OCT) scan (**b**) of an eye 3 months after XEN63 implantation with adjunctive Healaflow. The scan reveals a markedly elevated bleb, where the cavity appears spacious and diffuse, consistent with preserved outflow and minimal fibrosis. The presence of a low-reflectivity material within suggests residual cross-linked hyaluronic acid gel, contributing to sustained subconjunctival space maintenance.

No severe vision-threatening complications occurred in either group. In the early postoperative period, transient hypotony (IOP < 6 mmHg) was frequently observed; however, no severe cases occurred, and all episodes were generally mild and self-limiting. Shallow choroidal effusions were noted in three XENa eyes and two XENhf eyes, all of which resolved with conservative management. One eye in the XENa group also experienced anterior chamber shallowing, which gradually resolved during the first postoperative week without requiring surgical intervention. Additionally, one case of mild hyphema (<1 mm) was observed in the XENhf group, which resolved spontaneously within a few days. There were zero cases of bleb infection or endophthalmitis during follow-up. There was also no evidence of any inflammatory reaction or intolerance specifically attributable to the HF material, which was clinically evident in 80% of the eyes at 3 months (Figure 1a,b—showing bleb status at 3 months).

## 4. Discussion

While the XEN63 gel stent has demonstrated promising hypotensive efficacy with a more favorable safety profile compared to traditional trabeculectomy, it is not without its own postoperative challenges [3,8,9,10,11]. Despite its advantages, a significant proportion of patients still require postoperative interventions to maintain or restore adequate filtration. In real-world series, the need for postoperative needling or revision of XEN63 blebs to restore filtration is commonly reported, ranging from 10% to 41% [12,13,14,15,16,17,18]. This wide variability is likely due to differences in surgical technique, clinical indications for intervention, as well as variations in follow-up duration and protocols among studies. Therefore, despite its advantages, its success often relies on the early detection of filtration failure and timely interventions, leading some authors to consider needling not as an additional procedure, but rather as an integral part of the standard postoperative management of XEN, similarly to how laser suture lysis is regarded in trabeculectomy [3]. This need for frequent visits and interventions somewhat conflicts with the original goal of achieving a truly minimally invasive surgical approach. Hence, there is a growing need to identify strategies that can reduce the postoperative burden of XEN surgery, for both the patient and the physician.

HF is a cross-linked hyaluronic acid gel that has been investigated in conventional glaucoma surgeries with promising results. Its viscoelastic and space-maintaining properties are intended to reduce postoperative scarring and support the formation of a functional filtering bleb. HF typically remains present in the subconjunctival space for several weeks after the surgery, with studies reporting a persistence of its viscoelastic structure for up to 3 months. In nonpenetrating deep sclerectomy, implanting cross-linked HA under the scleral flap and subconjuctivally improved long-term filtration and IOP control [4]. In trabeculectomy, several studies have reported that adding HF can help maintain bleb function. A 5-year randomized trial in Chinese patients showed that trabeculectomy with a cross-linked HA implant achieved a lower mean IOP at 5 years and a higher complete success rate (78% vs. 54%) compared to trabeculectomy alone, with a better bleb morphology [19]. Similarly, Wu et al. (2021) found that adding HF to primary trabeculectomy resulted in more eyes with functional blebs and fewer scarring failures [6]. Not all studies have shown a significant benefit, however; Papaconstantinou et al. (2015) reported that at 6 months, trabeculectomy with HF had a safety profile similar to standard trabeculectomy, with no significant difference in IOP or bleb failure rates, possibly due to the relatively short follow-up or small sample [5]. Moreover, they did not use antifibrotics: we do not believe that HF can fully replace MMC; rather, we consider the two substances to act in a complementary manner. Additionally, differences in technique might play a role: insufficient or mis-targeted gel could reduce efficacy. In these studies, no adverse reactions attributable to HF were reported; in particular, there was no evidence suggesting that its long-term persistence could trigger inflammation or fibrosis.

Our study extends these findings to a minimally invasive surgery setting, indicating that even when a bleb is created through an ab interno XEN63 stent, the postoperative healing can be favorably altered by the presence of HF. Of note, both our study arms received MMC, which is a potent antifibrotic standard in XEN surgery, yet we still observed an additional benefit with HF. This suggests a complementary mechanism: MMC indiscriminately stops subconjunctival fibroblastic activity early on, whereas HA gel provides structural support and ongoing modulation of the wound environment, possibly sequestering growth factors like TGF-β and inhibiting fibroblast migration over time [6,20]. The combination of MMC plus HF may therefore achieve a more ideal balance for a longer period; actually, in 80% of patients in the XENhf group, a highly elevated translucent bleb—suggestive of persistent HF in the filtration area—was still visible at 3 months, as confirmed by AS-OCT imaging performed in a subset of patients. We speculate that a closed-conjunctiva approach, without surgical dissection of the conjunctival plane, may have contributed to even more prolonged in situ retention.

Although no statistically significant differences were found between the two groups regarding the success rate, IOP reduction, or number of medications, our results demonstrate that after 3 months, eyes that received HF required significantly fewer postoperative procedures (n = 2) compared to eyes that underwent XEN63 implantation alone (n = 8). Moreover, even in cases where an IOP elevation occurred, the needling procedure was generally more effective, suggesting that the fibrosis that developed was less severe and more limited in extent around the external ostium of the stent in the XENhf group. The usefulness of the addition of HF is an important finding for surgical practice: fewer needling procedures translate to less risk of infection and patient discomfort, as well as reduced healthcare costs and visits.

Nevertheless, both our study groups achieved a similar IOP reduction (38.8% and 31.8% in XENa and XENhf groups, respectively) and success rate from baseline, similarly to previously published studies on XEN63 [8,12,13,16,21,22].

Although the structural and isotonic properties of HF may promote a moderate and uniform aqueous humor outflow [6], buffering against excessive filtration and thereby reducing postoperative complications, our study did not show a lower incidence of hypotony and its related complications in the XENhf group, possibly due to the limited sample size. Nevertheless, such complications in XEN surgery tend to be less frequent and less severe than those typically observed with conventional trabeculectomy [9,10,16].

We acknowledge several important limitations of our study, including the modest sample size and the short follow-up period, which was limited to the immediate postoperative period; these factors may affect the statistical power, limit the generalizability of our findings, and prevent conclusions about long-term outcomes or complications. Nonetheless, we intentionally focused on the early postoperative window, which is clinically critical and when needling procedures are most frequently required [8,13]. A longer follow-up is needed to determine if the early advantage conferred by HF is sustained in the following years or if scarring eventually catches up once the gel is fully resorbed. Additionally, while our study was not randomized, the groups were significantly similar and surgeries performed in a standardized fashion. A randomized controlled trial would provide greater evidence and could also investigate patient-reported outcomes (bleb-related discomfort, recovery experience), which we did not systematically capture. Furthermore, prospective studies would allow for standardized imaging protocols to quantify bleb morphology (e.g., height, width, internal reflectivity) and enable correlation analyses between these structural parameters and clinical indices such as IOP or medication use. Such trials could also assess if lower MMC concentrations combined with HF can achieve similar success with fewer side effects. Finally, an interesting question is whether repeated injections of cross-linked HA at follow-up (after the initial gel degrades) could prolong bleb function further. This could be analogous to giving periodic 5-FU injections for bleb maintenance but using a viscoelastic approach.

## 5. Conclusions

Our study supports the non-inferiority of adjunctive HF use in XEN63 surgery compared to the standard technique, as we did not observe any statistically significant differences in IOP reduction, medication burden, or CDVA outcomes between the two groups. However, the use of HF seems to offer a clinical benefit by contributing to a reduction in the number of postoperative interventions required, as observed in our series. This translates into a clinically meaningful decrease in therapeutic burden for both patients and clinicians, minimizing the need for additional procedures, follow-up visits, and their associated risks. This advantage is particularly relevant in clinical settings where logistical constraints, such as limited access to operating rooms or minor procedure spaces, may hinder timely bleb revision. In such contexts, the inability to perform needling may lead to the premature reintroduction of topical therapy, thereby compromising the rate of complete surgical success. Moreover, these findings highlight a novel way to tackle the issue of bleb fibrosis in glaucoma surgery: by combining a minimally invasive stent with a bioengineered anti-scarring gel. This is, to our knowledge, the first comparative report on XEN63 with the HF adjunct, and it underscores the potential of biomaterials to improve outcomes in glaucoma surgery. If corroborated by larger and longer-term studies, adjunctive HF may become a valuable tool for surgeons to improve bleb survival and reduce the failure rate of XEN gel stents.

## Figures and Tables

**Table 1:** Demographic data of patients in the two groups (XENa and XENhf).

	XENhf (n = 10)	XENa (n = 10)	*p*-Value
Age (y), mean (SD)	69.7 ± 13.2	73.5 ± 11.8	0.49 *
Gender (M/F), n (%)	4 40%/6 (60%)	5 (50%)/5 (50%)	1.00 **
Preoperative IOP (mmHg), mean (SD)	21.9 ± 2.9	22.7 ± 3.2	0.56 *
Number of glaucoma medications, mean (SD)	3.2 ± 0.9	3.1 ± 0.8	0.81 ***
Glaucoma type (OAG/PXG), n (%)	8 (80%)/2 (20%)	7 (70%)/3 (30%)	1.00 **
Preop CDVA (logMAR), mean (SD)	0.12 ± 0.15	0.1 ± 0.13	0.57 ***
Lens status (phakic/pseudophakic), n (%)	0 (0%)/10 (100%)	0 (0%)/10 (100%)	1.00 **
Follow-up (months), mean (SD)	4.8 ± 1.5	4.4 ± 1.3	0.57 ***

XENa: XEN implant alone; XENhf: XEN implant + subconjunctival Healaflow; IOP: intraocular pressure; OAG: open-angle glaucoma; PXG: pseudoexfoliative glaucoma; CDVA: corrected distance visual acuity. *: *t*-test. **: Fisher’s exact test. ***: Mann–Whitney U test.

**Table 2:** Postoperative data at 3-month follow-up in patients treated with XENa and XENhf.

	XENhf (n = 10)	XENa (n = 10)	*p*-Value
IOP (mmHg), mean (SD)	14.8 ± 2.9	13.6 ± 2.1	0.31 *
Mean IOP (mmHg) reduction from baseline (mean absolute (SD) and percent)	7.1 ± 3.4 (31.8%)	9.1 ± 4.1 (38.8%)	0.25 *
Number of glaucoma medications, mean (SD)	0.1 ± 0.3	0.2 ± 0.4	0.73 **
Number of procedures (bleb needling or revision)	2	8	<0.05 ***
Patients with ≥1 needling, n (%)	2 (20%)	4 (40%)	0.63 ****
Postop CDVA (logMAR), mean (SD)	0.14 ± 0.11	0.13 ± 0.12	0.74 **

XENa: XEN implant alone; XENhf: XEN implant + subconjunctival Healaflow; IOP: intraocular pressure; CDVA: corrected distance visual acuity. *: *t*-test. **: Mann–Whitney U test. ***: Two-proportions z-test. ****: Fisher’s exact test.

## Data Availability

The data presented in this study are available on reasonable request from the corresponding author.
